# FDA launches health care at home initiative to drive equity in digital medical care

**DOI:** 10.1038/s41746-024-01198-2

**Published:** 2024-08-21

**Authors:** Stefanie Brückner, Celia Brightwell, Stephen Gilbert

**Affiliations:** 1 Else Kröner Fresenius Center for Digital Health, TUD Dresden University of Technology, Dresden, Germany; 2 Chair of Digital Cultures, Faculty of Linguistics, Literature and Cultural Studies, School of Humanities and Social Sciences, TUD Dresden University of Technology, Dresden, Germany

**Keywords:** Health policy, Health services

## Abstract

A highly ambitious FDA initiative will explore, through a hub and ideas lab, how equitable healthcare at home can be delivered, recognizing that this is unlikely to come about without intervention. Market forces, as shaped by current regulations, are leading to digital health tools developed and operating in islands rather than enabling integrated digital care. Can the initiative, which adopts system-level regulatory thinking, solve this issue?

Healthcare has been described as one of the ‘industries’ that has the greatest inertia, preventing structural and organizational change of service provision^1–3^. General system resistance to change, as well as many specific inertial factors relating to the adoption and integration of information technologies, has a profound influence on the potential for and the rate of adoption of new digital health concepts^4^. The COVID-19 pandemic resulted in a surge of interest and adoption of digital health, including telemedicine platforms^5^, and evidence is that these approaches have been ‘sticky’ (in the sense of interest and use being sustained, rather than use limited to the special circumstances of the pandemic) – although use has fallen back from the peak during the pandemic^6^. Healthcare regulation can serve as an inertial factor, and it has a profound impact on how technology is developed and how it can be usefully, sustainably, ergonomically, and transformatively integrated into the lives of patients in their environment^7^. There is good evidence that digital medicine technologies are not being coherently and holistically developed to meet these needs in an integrated fashion^8–10^, and this may not be something that can be resolved by individual businesses developing independently regulated products and operating according to pure capital market forces. Who will solve this problem? Enter the FDA.

## Regulation for the system and the care, not just the product

Is the role of a federal regulator to sit back, operate passively, clarify frameworks, publish guidelines for individual device types, provide authorizations, and initiate enforcement actions? Alternatively, is their role to take a helicopter view and to ask the fundamental (and maybe more important) questions: is the system functioning correctly, and how can the approval strategies of isolated new products, based on new technologies, incorporate the concept that they should all, somehow, work ‘holistically’ together to enable overall better healthcare delivery, experience, efficiency, and even equity?

Recently, the three US federal administrations responsible for the regulation of health have shown ambition to address the system as well as the individual product. The Federal Trade Commission (FTC) will likely have a major role in the proposed Algorithmic Accountability Act (2023)^11^, the Office of the National Coordinator (ONC) for Health Information Technology in the Department of Health and Human Services (HHS) has put in place system-level interoperability requirements^12^, and now, the FDA’s Center for Devices and Radiological Health (CDRH) has launched the Health Care at Home Initiative^13^. This is not a law, nor a rule, nor a guidance, nor an investigation of a new approach to regulation. While it may include aspects of the latter, it is an initiative and an exploration with the starting premises that the home environment can and will be an integral part of the healthcare system, and a critically important focus of future care delivery. The initiative addresses the fundamental questions of whether devices intended for use in the home, instead of for isolated independent functioning, can be designed to operate “as part of an integrated, holistic environment” that “seamlessly integrates into an individual person’s lifestyle” and use “medical-grade, consumer-designed, customizable technologies”^13^. Important considerations will be how home-based devices and platforms for integrated home care can be made resiliently cybersecure^14^ and how these devices can be approved and reimbursed as suites of devices^15^.

## Is seamless integrated home digital health possible and how can it be assessed and authorized?

The current product development pathways of companies, shaped by market forces and the policies of regulators, lead to the opposite of seamless and integrated sets of technologies and the result is the need for patients to “have to use several disparate medical devices, some never intended for the home environment”^13^. The FDA commissioner Robert Califf spoke about this challenge only two months before the launch of the Health Care at Home Initiative, saying “If you think about device development, like sensors, if you do it one at a time, you’ll never put a whole suite together into something that fits together in the [home] environment. [The FDA is] working on a strategy on how to create regulatory pathways to help make that happen”^16,17^. Califf’s insight into the challenges of building technologies that work together is not surprising, as between his former and current periods in office as the commissioner at the FDA, he served as an advisor to Alphabet (the Google, DeepMind, and Verily parent company)^18^.

The digital tech ecosystem, as clearly demonstrated by the products on our wrists and in our pockets, can produce seamlessly integrated and customizable technologies. The building of customizable interactions that enable suites of apps and wearables is harder to do in digital medicine, where products must go through regulatory authorization (including risk assessment, and validation of functionality, then clinical evidence generation), and there are no easy solutions – a challenge addressed by previous News & Views articles in the journal^15,16^. Indeed, regulatory guidance in the EU has imposed challenges to the concept on flexibly building together dynamic suites of devices^19^.

It is not fully described how the FDA initiative will address these challenges. The stated methods focus on bringing system-level human-centered design thinking, research on human-to-technology interactions, and technology-to-technology interoperability to bear, through an ideas lab, on the designed and pre-existing home environment^13^. They plan to do this in cooperation with expert agencies, and their ambition is demonstrated in the scope of the initiative. The FDA plans to consider how homes could be built to be suited to home healthcare, as well as how cooperative design of devices could be optimized. The stakeholders the FDA wants to influence through the initiative are interesting, as they include not only the developers, and the wider societal debate about care transformation, but also the care providers, who are less classically part of the FDA’s remit. One methodological focus of the hub is on the design and then use of an Augmented Reality/Virtual Reality (AR/VR)-enabled home prototype, to explore connected care.

## Is equity in healthcare at home possible?

The short press release announcing the initiative uses the word equity 9 times, including in the title as the prime goal of the entire activity. This is admirable and interesting, as the FDA has set out an ambition to address how care at home can be achieved in a manner that is equitable, that helps address unequal healthcare provision^20^, and avoids the much-discussed dangers of the ‘digital divide’^21–23^. The FDA’s initiative goes further than this though. They acknowledge in their press release that many aspects of traditional clinic-focused healthcare delivery are delivered in a manner that is highly inequitable, which disproportionately affects “people from various racial and ethnic minority populations and those who live in rural communities and lower-income neighborhoods”. They propose that digitally facilitated healthcare at home can be, if holistically promoted by regulators, a main arm of the solution “to meet the health care needs of millions of people who have no or limited access to health care systems.” To achieve its goal, however, the FDA’s approach to building AR/VR-enabled home prototypes would need to capture a vast array of different home configurations, living styles, and realities of care provision. Whilst AR/VR-based approaches have strengths, their application in the medical domain is not without challenges^24^ and it is to be hoped that the FDA and their partners will use multiple research modalities to address equity challenges and to explore how equity can be promoted through holistic and/or participatory design. Such approaches must take into account that technology is not always the desired or even the most effective solution to closing the gap between health disparities^25,26^ and can have implications for privacy^27^.

## A bold vision

One cannot challenge the boldness and timeliness of this vision. The degree to which regulators should shape rather than minimally police the market is a contentious issue. Arguably, existing regulations have already been shaping the market in its development tracks towards disparate unconnected solutions that serve neither the patients, healthcare systems, nor developers. The FDA acknowledges that to regulate is to shape and they now see that they must take action to shape for the better, acknowledging the need for the holistic development of digital medicine for the wider good. Time will tell how effective they will be – but it is to be celebrated that they have identified the challenge and are starting to address it. It remains to be seen whether the FDA will make use of international collaborations, e.g. through the International Medical Device Regulators Forum (IMDRF), to explore if progress can be made through shared learning, or whether they will foster parallel collaborative academic-led research in this area. The holistic development of at-home digital medicine is hard. There will not be many easy wins – simple standardization is easy, like the introduction of a compulsory common charger for mobile devices, to be enforced through regulation in the EU^28^. Health regulation and technology assessment needs to address cross-product approaches for evaluation alongside the flexible building of digital product suites – not an easy challenge^15,16,29^ – so hopefully the FDA is in this for the long term.

### Reporting summary

Further information on research design is available in the Nature Research Reporting Summary linked to this article.

### Supplementary information

Reporting Summary

## References

[1] Coiera, E. Why system inertia makes health reform so difficult. *BMJ* **342**, d3693 (2011). 21700652 10.1136/bmj.d3693

[2] Cohen, J. Inertia In American Healthcare: Muddling Along Inelegantly. *Forbes* https://www.forbes.com/sites/joshuacohen/2020/02/03/inertia-in-american-healthcare-muddling-along-inelegantly/ (2020).

[3] Hunter, D. J. & Bengoa, R. Meeting the challenge of health system transformation in European countries. *Policy Soc.* **42**, 14–27 (2023). 10.1093/polsoc/puac022

[4] Iyanna, S., Kaur, P., Ractham, P., Talwar, S. & Najmul Islam, A. K. M. Digital transformation of healthcare sector. What is impeding adoption and continued usage of technology-driven innovations by end-users? *J. Bus. Res.* **153**, 150–161 (2022). 10.1016/j.jbusres.2022.08.007

[5] Greenhalgh, T., Koh, G. C. H. & Car, J. Covid-19: a remote assessment in primary care. *BMJ* **368**, m1182 (2020). 32213507 10.1136/bmj.m1182

[6] Lee, E. et al. Updated National Survey Trends in Telehealth Utilization and Modality: 2021–2022 (Issue Brief No. HP-2023-09). Office of the Assistant Secretary for Planning and Evaluation, U.S. Department of Health and Human Services. (2023) 38913813

[7] Gilbert, S. et al. Learning From Experience and Finding the Right Balance in the Governance of Artificial Intelligence and Digital Health Technologies. *J. Med. Internet Res.* **25**, e43682 (2023). 37058329 10.2196/43682 PMC10148205

[8] Iyamu, I. et al. Challenges in the development of digital public health interventions and mapped solutions: Findings from a scoping review. *Digit Health* **8**, 20552076221102255 (2022). 35656283 10.1177/20552076221102255 PMC9152201

[9] Lehne, M., Sass, J., Essenwanger, A., Schepers, J. & Thun, S. Why digital medicine depends on interoperability. *npj Digit. Med.* **2**, 1–5 (2019). 31453374 10.1038/s41746-019-0158-1 PMC6702215

[10] Lancet, T. Making sense of our digital medicine Babel. *Lancet* **392**, 1487 (2018). 30496043 10.1016/S0140-6736(18)32545-5

[11] US Congress. Text - H.R.5628 - 118th Congress (2023-2024): Algorithmic Accountability Act of 2023. https://www.congress.gov/bill/118th-congress/house-bill/5628/text (2023).

[12] Office of the National Coordinator for Health Information Technology (ONC) of the US Department of Health and Human Services. HHS Finalizes Rule to Advance Health IT Interoperability and Algorithm Transparency. https://www.hhs.gov/about/news/2023/12/13/hhs-finalizes-rule-to-advance-health-it-interoperability-and-algorithm-transparency.html (2023).

[13] FDA. FDA Launches Health Care at Home Initiative to Help Advance Health Equity. https://www.fda.gov/medical-devices/medical-devices-news-and-events/fda-launches-health-care-home-initiative-help-advance-health-equity (2024).

[14] Gilbert, S., Ricciardi, F., Mehrali, T. & Patsakis, C. Can we learn from an imagined ransomware attack on a hospital at home platform? *npj Digit. Med.* **7**, 1–5 (2024). 38509171 10.1038/s41746-024-01044-5 PMC10954698

[15] Mathias, R., McCulloch, P., Chalkidou, A. & Gilbert, S. Digital health technologies need regulation and reimbursement that enable flexible interactions and groupings. *npj Digit. Med*. **7**, 148 (2024). 38890404 10.1038/s41746-024-01147-z PMC11189410

[16] Mathias, R., McCulloch, P., Chalkidou, A. & Gilbert, S. How can regulation and reimbursement better accommodate flexible suites of digital health technologies? *npj Digit. Med.* **7**, 170 (2024). 38956279 10.1038/s41746-024-01156-y PMC11219713

[17] Al-Faruque, F. Califf: New systems needed for bringing rare disease treatments, at-home devices to market. *Regulatory Affairs Professionals Society* https://www.raps.org/news-and-articles/news-articles/2024/2/califf-new-systems-needed-for-bringing-rare-diseas (2024).

[18] McGinley, L. & Roubein, R. Califf narrowly confirmed for FDA commissioner, providing beleaguered agency with its first permanent chief in 13 months. *Washington Post* https://www.washingtonpost.com/health/2022/02/15/califf-fda-commissioner/ (2022).

[19] MDCG. MDCG 2023-4. Medical Device Software (MDSW) – Hardware combinations Guidance on MDSW intended to work in combination with hardware or hardware components. https://health.ec.europa.eu/system/files/2023-10/md_mdcg_2023-4_software_en.pdf (2023).

[20] Wilkins, C. H. Unequal Treatment: Disparities in Care Continue. *NEJM Catalyst* **5**, 10.1056/CAT.24.0207 (2024).

[21] Budd, J. et al. Digital technologies in the public-health response to COVID-19. *Nat. Med*. **26**, 1183–1192 (2020). 32770165 10.1038/s41591-020-1011-4

[22] Saeed, S. A. & Masters, R. M. Disparities in Health Care and the Digital Divide. *Curr. Psychiatry Rep.* **23**, 61 (2021). 34297202 10.1007/s11920-021-01274-4 PMC8300069

[23] Mitchell, U. A., Chebli, P. G., Ruggiero, L. & Muramatsu, N. The Digital Divide in Health-Related Technology Use: The Significance of Race/Ethnicity. *Gerontologist* **59**, 6–14 (2019). 30452660 10.1093/geront/gny138

[24] Freyer, O. & Gilbert, S. Bridging between hype and implementation in medical extended reality. *npj Digit. Med.* **6**, 1–3 (2023). 38062115 10.1038/s41746-023-00972-y PMC10703922

[25] Yao, R. et al. Inequities in Health Care Services Caused by the Adoption of Digital Health Technologies: Scoping Review. *J. Med. Internet Res.* **24**, e34144 (2022). 35311682 10.2196/34144 PMC8981004

[26] The Lancet Digital Health. Digital technologies: a new determinant of health. *Lancet Digit Health* **3**, e684 (2021). 34711372 10.1016/S2589-7500(21)00238-7

[27] Sekalala, S., Dagron, S., Forman, L. & Meier, B. M. Analyzing the Human Rights Impact of Increased Digital Public Health Surveillance during the COVID-19 Crisis. *Health Hum. Rights* **22**, 7–20 (2020). 33390688 PMC7762901

[28] News - European Parliament. Long-awaited common charger for mobile devices will be a reality in 2024. https://www.europarl.europa.eu/news/en/press-room/20220930IPR41928/long-awaited-common-charger-for-mobile-devices-will-be-a-reality-in-2024 (2022).

[29] Welzel, C. et al. Holistic Human-Serving Digitization of Health Care Needs Integrated Automated System-Level Assessment Tools. *J. Med Internet Res.* **25**, e50158 (2023). 38117545 10.2196/50158 PMC10765286

